# Biosphere Plastic Contamination and Microbial Alternatives for a Sustainable Degradation of Plastic Waste

**DOI:** 10.3390/microorganisms13061246

**Published:** 2025-05-28

**Authors:** María Elena Báez-Flores, Martín Ernesto Tiznado-Hernández, Martina Hilda Gracia-Valenzuela, Rosalba Troncoso-Rojas

**Affiliations:** 1 Facultad de Ciencias Químico-Biológicas, Universidad Autónoma de Sinaloa, Ciudad Universitaria, Av. de Las Américas Esq. Josefa Ortiz de Domínguez S/N, Culiacán CP 80013, Mexico; 2 Coordinación de Tecnología en Alimentos de Origen Vegetal, Centro de Investigación en Alimentación y Desarrollo, Asociación Civil, Carretera Gustavo Enrique Astiazarán Rosas No. 46, Col. La Victoria, Hermosillo CP 83304, Mexico; tiznado@ciad.mx; 3 Instituto Tecnológico del Valle del Yaqui, Tecnológico Nacional de México, Av. Tecnológico, Block 611, Bácum CP 82276, Mexico; martina.gv@vyaqui.tecnm.mx

**Keywords:** plastic contamination, plastic degrading microorganisms, plastic biodegradation, bacterial bioplastics, sustainability

## Abstract

In the mid-twentieth century, the solid waste generated was mostly made of biodegradable materials. However, the invention of plastic and its widespread use have led to a staggering accumulation of plastic in the environment, posing a severe threat to the biosphere. The environmental degradation of plastic can take thousands of years and poses a significant concern for environmental and human health. Until recently, it was thought that some plastics were non-biodegradable; however, there are microorganisms capable of degrading both plastics derived from fossil resources and those from biomass or renewable resources. This review aims to highlight the impact of plastic waste on the environment and the biosphere, as well as the great taxonomic diversity of microorganisms potentially linked to plastic degradation. Research in plastic biodegradability includes the identification of bacteria, fungi, archaea, and algae from virtually any environment: soil, atmosphere, landfills, freshwater, seawater, marine sediments, rumen, and waxworm guts. Identifying microbial consortia that degrade plastic and improving their degrading activity could shorten the plastic degradation time and reduce its uncontrolled accumulation around the globe. Research in this field is vital for advancing biodegradable plastics and elucidating the potential and limitations of microbial degradation as a large-scale approach to plastic pollution.

## 1. Introduction

Plastic is one of the best-known and widely used materials. They are synthetic materials obtained by polymerizing carbon atoms to obtain molecular chains of organic compounds. Plastics became popular due to their valuable properties, e.g., the ease with which they can be molded, their impermeability, low density, low electrical conductivity, corrosion and outdoor resistance, resistance to various chemical and biological factors, and low cost [1]. These properties have made plastics one of the favorite materials in the food, pharmaceutical, medical, cosmetic, automotive, recreational, and construction industries. Global plastic production has been increasing, especially over the past 50 years. According to the Plastics Europe Association, in 2022, global plastic production reached 400.3 million tons. Europe had a global plastic production of 14%, while North America and China accounted for 17% and 32%, respectively. Likewise, the rest of Asia produced 24% of the plastic. In contrast, the global production of post-consumer recycled plastics in 2022 was 35.5 million tons, with North America contributing 8%, Europe 21%, and China 24%. It is worth mentioning that the rest of Asia contributed 34% [2]. The wide use of plastic has caused its accumulation in the environment, representing a severe problem for the biosphere. Current disposal methods for plastic waste include landfilling, incineration, and mechanical and chemical recycling [3]. Today, only 9–12% of global plastic waste is reported to be recycled and incinerated. In comparison, up to 79% is disposed of in landfills or the natural environment, indicating a great need to explore innovative recycling methods to eliminate plastic waste [4].

Plastics, microplastics, and nanoplastics are found in soil [5,6], air [7], surface waters, sediments, the marine environment [8,9,10], including the polar ice caps [11,12], as well as in the bodies of organisms from aquatic and terrestrial environments [13]. Even microplastic beads used in the cosmetics industry account for a significant proportion of plastic waste in aquatic environments [14,15,16]. Unfortunately, this contamination goes beyond the environmental sphere, reaching human beings. The presence of microplastics in blood [17] and human placenta has been reported [18,19]. Furthermore, primary human monocytes and dendritic cells have been shown to internalize nanoplastics, e.g., poly(vinyl chloride) (PVC) and poly(styrene) (PS), which subsequently leads to the secretion of both pro- and anti-inflammatory cytokines [20].

Recent studies have definitively demonstrated [5] that certain plastics are indeed biodegradable. A diverse range of microorganisms can effectively break down plastics sourced from both fossil fuels and renewable biomass. This review will highlight the impact of plastic waste on the environment, diverse aspects of the biodegradability of various types of plastics, and the taxonomic diversity of microorganisms from the Bacteria, Archaea, and Eukarya domains involved in plastic degradation, including the already known and those unexpectedly linked to the degradation of plastics.

## 2. Plastics in the Biosphere: Outlining the Problem

The intensive use of these materials in industries such as food, transportation, construction, clothing, medical, personal care products, and recreational industry, combined with its high stability, has caused severe environmental pollution problems [21,22,23]; nowadays, plastics are widely distributed even in remote environments, e.g., the atmosphere, the deep-sea floor, polar waters, and marginal seas [24,25].

The waste generated by human activity until the mid-twentieth century consisted mainly of biodegradable waste [1]. Currently, discarded waste worldwide reaches 50 to 80% of waste floating on the ocean surface and seabed, contaminating mainly the gyres and oceanic convergences [26]. In 2020, world plastic production reached 375.5 million tons. From that, 29.5 million tons were produced in Europe, where 23% of the post-consumed plastic was disposed of in landfills; 42% was used for energy recovery, and 35% was recycled [27].

However, an analysis of all plastics ever produced [28] showed that in 1950, plastic production accounted for 2 million metric tons, and it is estimated that by 2015, 8300 million metric tons of plastic had been produced, generating about 6300 million metric tons of plastic waste. Of this, 9% was recycled, 12% was incinerated, and 79% was landfilled or thrown off in natural environments. Calculations predict that if plastic production and waste management trends continue, 12,000 million metric tons will be in landfills or the natural environment by 2050 [28].

Considering that most conventional plastics are “non-biodegradable,” some strategies have been proposed to minimize the impact of these materials on ecosystems. Among the most important strategies are incineration, recycling, and producing plastics with a high degree of biodegradability. Incineration is used to remove petroleum-based plastics, but it is a very polluting process, increasing the atmospheric CO_2_ and releasing hazardous substances such as dioxins, furans, and hydrogen chloride [29,30]. The production and incineration of plastic generated about 850 million metric tons of greenhouse gases (GHG) in 2019, accounting for 2.6% of the global energy from GHG emissions [31]. Not all plastics are recyclable; thermoplastics can be recycled, while thermo-stable plastics, which develop irreversible changes during molding, cannot be recycled [1]. The ecological problem posed by synthetic non-degradable polymers led to the development of biodegradable polymers. The term “degradation” describes any physical or chemical change in a material caused by environmental, chemical, or biological factors [1,22]. Further, the term “biodegradable plastic” refers to a plastic that microorganisms can degrade [32].

The term “bioplastic” refers to “either biodegradable plastics produced from fossil materials or bio-based plastics synthesized from biomass or renewable resources” [33]. According to European Bioplastics, “bioplastics” refers to biodegradable, bio-based polymers, or both. However, the term “biobased” is not equivalent to “biodegradable” since biobased bioplastics can be either biodegradable, e.g., poly(lactic acid) (PLA), non-biodegradable [bio-poly(ethylene) (bio-PE), bio-poly(propylene) (bio-PP), bio-poly(amide) (bio-PA), bio-poly(ethylene terephthalate) (bio-PET), and others [27,34]. Almost 50% of bio-based bioplastics are non-biodegradable [35]. The two leading bio-based plastics used are PLA (derived from sugar cane or corn starch) and PHAs (polyhydroxyalkanoates, produced by microorganisms from food, crops, or oil residues). Even when PLA is biodegradable, it does not naturally degrade in landfills but must be sent to industrial composting facilities, where it can be thermally treated to allow for microbial degradation. Indeed, biobased plastics are more expensive than conventional ones and may not be as environmentally friendly as expected [31,35].

### 2.1. Most Used Plastics Around the World

Considering all the available plastics, among the most widely used are the biodegradable aliphatic polyesters derived from fossil resources, including poly(ethylene adipate) (PEA), poly(ε-caprolactone) (PCL), poly(β-propiolactone) (PPL), poly(butylene succinate) (PBS), poly(ethylene succinate) (PES) polymers, and the aliphatic–aromatic copolyesters (AAC’s). Other widely used biodegradable aliphatic polyesters from renewable resources are Poly(3-hydroxybutyrate) (P3HB, belonging to the PHA group) and PLA [1,33]. Also, the synthetic polycarbonates, polyurethanes, polyamides (Nylon 6 and 4), copoly(amide-*co*-ester)s (CPAE), poly(ethylene) (PE), poly(ethylene terephthalate) (PET), poly(propylene) (PP), and poly(styrene) (PS) are of massive use. Additionally, the use of blends of polyesters with other polymers, e.g., PET and poly(butylene terephthalate) (PBT), is frequent, both previously considered non-biodegradable. Likewise, using polymer blends with starch is remarkable, e.g., PCL mixture with corn or tapioca starch [27,33].

#### Plastic Degradation Is a Significant Concern in Environmental and Human Health Scenarios

Conventional synthetic plastic degradation could take hundreds, even thousands of years [26,35]. Moreover, the “degradation” of these plastics only generates smaller particles, which are no longer evident but persist in the ecosystems. The MPs (100 nm to 5 mm) and nanoplastics (1 to 100 nm) [23] accumulated in the seas are vectors for major ocean pollutants and participate in their bioaccumulation in the marine environment and food webs [36,37]. About 14 million tons of microplastics are estimated to enter the ocean annually [38]. Even microplastic beads used in cosmetics and personal care products are one of the primary sources of potentially dangerous plastic material released into the marine environment [14,16]. In sand beaches and estuaries, the micro fragments of acrylic, PP, PE, polyamide, polyester, and poly(methyl methacrylate) are in concentrations of 3 to 30 kg/km^2^, increasing yearly. In the past decades, the amount of microplastics in the North Pacific Ocean has tripled, while near the Japanese coast, it has multiplied by ten every 2 or 3 years [1]. In addition, the plastic waste in the oceans in various concentrations and particle diameters [1,39] represents a danger to marine organisms that can be damaged by ingestion and suffocation. Further, planktonic and filtering organisms ingest plastics that stay trapped in their tissues [1]. Over 260 species have been reported to ingest or become entangled in plastic debris, which could result in impaired movement and feeding till death [13]. In bird species, up to 80% of individuals contain plastics in their stomachs [1]. Studies in the shrimps’ gastrointestinal tract (SGT) reported MPs in *Metapenaeus monoceros*, *Parapeneopsis stylifera*, and *Penaeus indicus*, impacting food safety and human health. The dominant MPs range from 100 to 250 μm [40], while in *Litopenaeus vannamei* and *Macrobrachium rosenbergii*, the MPs’ size ranged from 500 to 1000 µm [41]. SGT mainly contains MPs in the form of fibers, fragments, pellets, beads, and films. Up to six types of plastic polymers were identified in SGT [40]. These MPs in aquacultural products have several consequences for human health; e.g., the reduction in digestive enzyme activity and the exposure to MPs’ organic additives are able to cause reproductive toxicity, teratogenicity, and mutagenicity [42].

Some calculations estimate that 268,940 tons of plastic particles are floating in the Earth’s oceans [8]. In 2015, up to 99 million metric tons of MP debris were generated in the oceans from marine plastic litter (MPL) [43]. However, studies quantifying MPs in the oceans detected only 1% of the expected volume according to the estimated quantity of plastic in the oceans, reflecting a loss of 99% of MPs in the world’s oceans, whose fate is unknown [8,44]. In 2010, up to 12.7 million metric tons were estimated to arrive in the ocean [45]. Other calculations estimate more than 150 million tons of plastic in the oceans. Furthermore, it was predicted that by 2025, the oceans will contain one ton of plastic for every three tons of fish, and by 2050, it is envisioned that there will be more plastic than fish by weight [46]. Some researchers suggest that MPs can enter the atmosphere and be carried by the wind [47]. In the marine environment, waves can release MPs into the air through sea aerosols [48]. Additionally, roads contribute to airborne microplastics as plastic from tires and debris on the road surface break down into fine dust [49].

Atmospheric deposition of MPs has been detected even in remote regions. A study conducted in the Pyrenees reported a deposition rate of 365 MPs particles/m^2^/day and identified potential sources up to 95 km from the study site. The authors suggested a link between precipitation, wind speed and direction, and MP deposition [47]. Another study quantifying MPs in the atmosphere reported MPs in 98% of samples obtained by dry and wet deposition (due to gravity and rain, respectively). Calculations estimated a total deposition rate between 1000 and 4000 metric tons annually. A total of 70% of the atmospheric MPs were fibers, possibly from textiles; 30% were brightly colored microbeads, probably derived from paints and coatings. Dry-deposited MPs were smaller and more prevalent at high elevations, indicating that these MPs traveled higher in the atmosphere and probably traveled between continents [49]. This is of concern since airborne particulate matter affects the Earth’s climate through the absorption and dispersion of radiation [50], which is assessed by the effective radiative forcing (ERF) metric. Calculations revealed a small ERF for MPs compared to the total ERF attributed to aerosol–radiation interactions. However, the abundance and ERF of MPs will continue to increase [50] along with spillover effects on Earth’s climate. Findings of a pilot study performed on the French Atlantic coast provided the first evidence that MPs can be emitted from the ocean into the atmosphere through sea spray, driven by bubble bursting and wave action. The authors reported MPs in marine air samples, with higher concentrations during onshore winds and sea mist events (up to 19.4 MP/m^3^). Their findings suggest that the ocean may act not only as a sink but also as a secondary source of atmospheric MPs, potentially contributing to regional or even global air transport of MPs [48]. Regarding airborne MPs from tires on roads, in 2018, road transport accounted for 13% of Austria’s total suspended particulate emissions (TSP), with approximately 72% of these emissions attributed to non-exhaust sources, e.g., not related to fuel combustion, including tire, brake, and road surface wear. Among them, tire wear particles (TWPs) represent about 3.1% of the national TSP, highlighting their relevance as a microplastic emission source. These particles not only contribute to air pollution but also accumulate in terrestrial and aquatic environments [51]. In human toxicology, biomonitoring demonstrated that some chemicals used in manufacturing plastics, such as phthalates and bisphenol A (BPA), leach from standard plastic products, reaching human tissues by ingestion, inhalation, and dermal contact. Phthalates and BPA are detectable in the aquatic environment, dust, and air. Experimental evidence shows the correlation between the presence of these chemicals and several adverse health effects, mainly reproductive abnormalities. In adults, a negative association was reported between phthalates and semen quality, as well as between high exposures to phthalates and free testosterone levels. Moreover, a significant relationship was found between urine levels of BPA and cardiovascular disease, type 2 diabetes, and liver enzymes. There is evidence that low doses of BPA cause disruption of hippocampal synapses, leading to the appearance of a senile brain both in rats and monkeys. Additionally, BPA affects brain development, causing the loss of sex differentiation in brain structures and behavior [13]. Leslie and coworkers [17] hypothesized that exposure to plastic particles, present ubiquitously in the environment and the food chain, causes their absorption into the bloodstream. Unfortunately, the elimination rate of some plastic particles from the human body could be slower than their absorption rate [17]. On the other hand, MPs cross the placental barrier [18,19]. PEG fragments were found in the chorioamniotic membrane and the placental cotyledons [52]. A study detected MPs in human placentas and reported a correlation between MPs and ultrastructural alterations in some organelles, such as mitochondria and endoplasmic reticulum in placental cells. The authors hypothesized that MPs could cause epigenetic changes leading to phenotypic alterations in the long term [19]. Furthermore, Weber and coworkers reported that nanoplastics (PVC and PS) cause the release of cytokines in human monocytes and dendritic cells, inducing a pro-inflammatory response [20]. Bioaccumulation of MPs has been demonstrated in the liver, kidneys, brain [53], coronary and carotid arteries with atherosclerotic plaques, and plaque-free aortas [54]. MPs have also been detected in atheromatous plaques and patients with cardiovascular events [55]. Notably, one study reported significantly higher concentrations of MPs in normal brain samples (between 7 and 30 times greater) compared to liver or kidney samples. Furthermore, brain samples from individuals with a documented diagnosis of dementia showed even higher concentrations of MPs [53]. Additionally, MP concentrations were significantly higher in coronary and carotid arteries with atherosclerotic plaques than in non-atherosclerotic aortas, suggesting a potential association between MPs and atherosclerosis in humans [54]. In another study, patients with carotid artery plaques containing MPs had an increased risk of suffering a combination of myocardial infarction, stroke, or any cause of death at 34 months of follow-up compared to those without detectable MPs [55]. These findings indicate a possible adverse impact of MPs on humans’ neurological and cardiovascular health. A recent study suggested that starch-based microplastics (SMPs) may be as toxic as petroleum-derived plastics. Researchers observed SMP accumulation in the liver, intestines, and ovaries in murine models, accompanied by microstructural lesions. They also reported hyperglycemia, hepatic oxidative stress, lipid metabolism disruption, and alterations in the intestinal microbiota, including enrichment of metabolic pathways associated with insulin regulation and circadian rhythms. These findings underscore the need for more rigorous toxicological assessment before SMPs are widely used in food-related applications [56].

Given the current ubiquitous presence of plastic in nature, this material may become the geological signature of our era. Scientists are intensifying efforts to harness biological tools to break down and remove these polymers from the environment [57]. Recent research on the biodegradability of plastics in both soil and aquatic ecosystems has led to the identification of various microbial taxa capable of degrading synthetic polymers, including actinomycetes, fungi, archaea, algae, and even ruminal and gut-dwelling nematode-associated microorganisms. The finding and improvement of plastic-degrading microbial consortia and advances in genetic engineering to enhance their enzymatic activity represent promising strategies for accelerating plastic degradation and reducing its uncontrolled accumulation in terrestrial and aquatic environments worldwide.

## 3. Biodegradability of Plastics and Factors Affecting the Degradation Process: An Overview

Due to the recent invention of plastics, researchers thought that evolution had not designated enzymes capable of degrading them. Since the early 1970s, some authors have reported that several natural lipases, which hydrolyze fats and oils, can also hydrolyze the ester bonds of aliphatic polyesters. However, the aromatic polyesters remained in the status of “inert” against biological attack. Because of their insolubility in water and the size of the polymer molecules, microorganisms cannot uptake them. Therefore, they must excrete enzymes that hydrolyze them for degradation by the microbial cell metabolic pathways [32]. Microorganisms degrade polymers through a series of distinct, sequential stages: bio-deterioration (in which the chemical and physical properties of the polymers are altered), bio-fragmentation (where polymers are broken down into smaller, simpler molecules via enzymatic cleavage), assimilation (where microorganisms incorporate these smaller molecules), and mineralization (the final stage, in which oxidized metabolites are produced under either aerobic conditions (H_2_O, CO_2_) or anaerobic conditions (H_2_O, CH_4_, CO_2_) [58]. Microorganisms can produce assimilable molecules using polymers as the sole source of carbon. *Rhodococcus ruber* (C208), *R. rhodochrous* ATCC29672, and *Cladosporium cladosporoides* ATCC 20251 produce polysaccharides and proteins by using PE as a carbon source, while *Nocardia asteroides* GK911 produces proteins only. Other reported products of microbial degradation of polymers are fatty acids, aldehydes, carboxylic acids, ketones, alkenes, alcohols, phenols, esters, ethers, benzene, tetrachloroethylene, 1-monanalinoeoglycerol trimethylsilyl ether, betamethasone acetate, and azafrin, among others [59]. Finally, due to the polymer degradation process, water, carbon dioxide (aerobic degradation), methane (if anaerobic degradation), and new biomass are produced [32].

Among the factors influencing polymer degradation are surface conditions (surface area, hydrophilic and hydrophobic properties of the polymeric surface), first-order structures [chemical structure, molecular weight (MW), and MW distribution], as well as other properties such as glass transition temperature, melt temperature, modulus of elasticity, crystallinity, and crystal structure [33]. Other biological degradation factors include the functional groups (carbonyl, ester, vinyl) and double bonds on the polymer’s surface. Polymer incubation with microorganisms causes changes in the concentrations of these functional groups (consumption or production) depending on the balance of oxidation and degradation rates, which, in turn, is due to the nature of the microbial community on the polymer’s surface. Studies with PE showed that the major limitation in the attachment of the microorganisms to the PE is its relatively high hydrophobicity, in contrast with the hydrophilic surface of most microorganisms. In PE biodegradation, two main biochemical reactions occur: the reduction in its MW and the oxidation of the molecules (formation of carbonyl groups). The first is required to transport PE molecules through the cell membrane since microorganisms’ enzymatic systems can attack polymer chains ranging from 10 to 50 carbons. After the molecule’s size reduction, oxidation transforms the hydrocarbon into a carboxylic acid, which can be metabolized through β-oxidation and the Krebs cycle pathways [22]. Some microorganisms degrade polymers via biofilm formation. This boosts the degradation efficiency continued by the mineralization process. *P. aeruginosa* conducts biofilm formation, helped by alginate-like chemicals and quorum-sensing signaling systems (Rh1I/RhR), while *R. ruber* colonizes and degrades PE by forming biofilm and producing hydrolytic enzymes [58]. However, recent critical reviews question the validity of studies reporting PE biodegradation due to the lack of direct and quantitative evidence of mineralization. Such studies often rely on indirect indicators such as weight loss, surface erosion, or changes in functional groups, which may result from environmental weathering or partial oxidation rather than microbial assimilation and degradation into CO_2_ or biomass. According to the author, irrefutable evidence would require isotope-labeled polymers and rigorous controls, which are rarely applied [60].

Research in recent decades revealed that aliphatic polyesters are degraded by lipase, whereas aromatic polyesters are biologically inert. It was argued that the ester bonds neighboring bulky aromatic groups are less accessible to lipases. Also, it was noted that the chemical structure around the ester bond is not the main factor controlling these polyesters’ degradability but the material’s polymeric nature. It was observed that the rate of biodegradation (mmol/h) of various aliphatic polyesters with lipase from *Pseudomonas* sp. corresponded with *ΔTmt*, which represents the difference between the temperature at which degradation took place and the melting temperature (Tm) of the polyester. Since the Tm of a semi-crystalline polymer is related to its crystalline fraction, *ΔTmt* has been interpreted as a measure of the ability of the polymer chains to temporarily leave the order of polymer crystals and form a loop capable of penetrating into the active site of lipase localized on the surface of the material. Furthermore, the mobility chains model explains the non-degradability of PET and PBT, which have a high melting point of 200 °C [32]. Moreover, a high MW reduces the susceptibility to biodegradation. Indeed, it was reported that the PCL with a higher MW is degraded more slowly by a lipase from *Rhizopus delemar* than the lower MW PCL. Moreover, the degree of crystallinity affects degradation because enzymes primarily attack the amorphous domains of a polymer. This is because the molecules in the amorphous region are far apart, making them more susceptible to degradation. Therefore, the crystalline part of the polymer is more resistant to degradation than the amorphous region. Also, a high Tm reduces the biodegradability of the polymer [33].

Another factor affecting plastics biodegradation is the type of additives used in their manufacture, which could define the profile of microorganisms colonizing the polymer. De Tender and coworkers [9] reported more than 250 chemical compounds in the marine plastic litter (MPL), showing that pigment content on plastics can determine the bacterial community profile of MPL. The plastic’s microbial community composition significantly differed from that of sediments and seawaters [9]. Indeed, plastic debris is considered a different habitat denominated “The Plastisphere” [61]. Finally, environmental factors such as temperature, pH, salinity, oxygen levels, sunlight, water activity, stress, and culture conditions affect polymer degradation and define the microbial population and enzyme activity [59].

## 4. Diversity of Fungi and Bacteria Capable of Degrading Plastics

From the 1970s to now, researchers have reported the ability of microorganisms to degrade synthetic plastics. A study summarized several research findings in the plastic microbial degradation field [33]. In research about PU biodegradation using DGGE, it was reported that 37% and 45% of cultivable fungi in acidic and neutral soils degraded PU [62]. *Phoma* sp. (neutral soil) and *Geomyces pannorum* (acidic soil) were the primary fungi recovered by buried PU cultivation, representing more than 80% of the isolates. The authors argued that PU has analogous molecular bonds to those found in biological macromolecules and that fungal genes encode several hydrolases, increasing the probability of random PU degradation. Their result suggested that the presence of polymers in the environment induces the expression of these genes [62].

Méndez and coworkers [63] evaluated the degradative activity of fungi recovered from damaged PE objects obtained in a landfill. PE “pellets” were cultured in a minimal medium (MM). At 30 °C and pH 6.5, the biodegradation process of PE was slightly higher for *Aspergillus flavus*. Other fungi degrading PE were *Penicillium* sp., *P. implicatum*, *Helmintosporium* sp. 001, and *A. niger*. This study proved that the availability of an alternative carbon source, the pH, and temperature conditions determine the expression of the fungal enzymes, allowing for PE degradation [63]. Another research identified five soil micromycete species involved in PHA biodegradation [64]. Also, the review by [22] compiled evidence of nine fungal genera capable of degrading PE. In addition, Krueger and coworkers listed several microorganisms involved in conventional plastic degradation, including the basidiomycete *Trametes versicolor* [65]. Also, the Ascomycete *Chaetomium globosum* ATCC 16021 demonstrated the ability to degrade PCL [66]. Table 1 and Table 2 show the diversity of microorganisms reported in the above-cited works. *Nocardiopsis aegyptia*, a halophilic actinomycete from marine sediment, degrades P3HB (using it as the sole carbon source) with an extracellular depolymerase. A maximum percentage of weight loss (89.94%) was observed after 30 days of incubation. The authors reported that a seven-day culture stimulated degradation, suggesting a constitutive expression of the depolymerase, in contrast to other bacterial P3HB depolymerases, whose expression is repressed if a soluble carbon source is available [67]. Various actinomycetes and fungi are reported as PBS, PCL, and PLA degraders [66,68,69,70,71]. They are included in Table 1.

Bacteria also carry out PU degradation. Deteriorated rubber foam samples from a landfill were grown in MM, containing only mineral salts and commercial surface-coating-PU (hydroform, PUh) as a carbon source. The MM-PUh culture isolated *Alicycliphilus* sp. and *A. denitrificans*. PUh is a polyester containing N-methyl pyrrolidone (NMP) as an additive. The identified bacteria used NMP to grow in MM supplemented with NMP. Given the toxicity of NMP, this may help prevent its release into the environment. *Alicycliphilus* strains showed esterase activity in MM-PUh when the NMP substantially decreased in the medium. When *Alicycliphilus* was grown in LB broth, no such activity was detected, suggesting that the medium chemical composition induced the esterase activity. The NMP provides an easily utilizable carbon source; after that source is depleted, esterase activity is induced, and the bacteria degrade the PU [72]. De Tender and colleagues [9] studied the bacterial communities on marine plastic litter (MPL) by 16S metabarcoding. They found *Mycobacterium frederiksbergense* in high abundance (21% to 29%) on blue and yellow beach pellets but not in other plastics. This bacterium can metabolize polycyclic aromatic hydrocarbons like anthracene, which is used to produce anthraquinone, whose derivatives are, in turn, used for coloring resin pellets. The authors assumed that dyes in the plastic could attract bacteria that are able to metabolize those compounds. For instance, the *Vibrionaceae* or *Pseudoalteromonadaceae* are commonly found in MPL but rarely observed in seawater or sediments [9].

The PHA degradation research has reported diverse microorganisms decomposing PHAs. The concentration of these bacteria in garden soil ranges from 3 × 10^5^ CFU/g [1] to 1 × 10^14^ CFU/g of soil [73]. However, a PHA bottle would take approximately three months to degrade. These bacteria are also found in lakes, marine environments, and sludge from wastewater treatment plants [1]. *Firmicutes* and *Proteobacteria* degrade PHA, PCL, and PBS but not PLA [74]. A detailed list can be reviewed in Table 1 [74]. On the other hand, PCL-degrading bacteria have been reported at sea depths over 5000 m [75]. A study on PHA biodegradation identified microbial populations in soils dominated by 19 bacterial species, e.g., *Chromobacterium violaceum*, *Arthrobacter artocyaneus*, and *Cupriavidus gilardii*. The complete list is presented in Table 2. The bacterial concentration influenced the velocity of PHA degradation. Also, the total counts of microorganisms from the biofilm on the PHA surface were one or two orders of magnitude higher than in the control soil [64].

By Next Generation Sequencing (NGS), researchers studied the PE degradation using a consortium of indigenous marine communities (INDG) and another one of indigenous communities supplemented with *Lysinibacillus* sp. and *Salinibacterium* sp. (BIOG) [76]. The BIOG biofilm bacteria decreased the PE weight by 19%, while the INDG biofilm caused a weight loss of 4.2%. *Proteobacteria* dominated all the bacterial communities, and the *Alphaproteobacteria* and *Gammaproteobacteria* were the most abundant classes in the PE biofilm. In the mature biofilm, the authors found a significant increase in *Bacillus* sp. and species degrading hydrocarbons or natural polymers, e.g., *Pseudonocardia* carrying the gene encoding the AlkB-rubredoxin fused proteins, a critical enzyme in the alkane bacterial degradation, and the genus *Cellulosimicrobium* integrated for hydrocarbon and cellulose degraders. Also, the last two genera showed higher abundances in the BIOG community, which is in agreement with the higher concentration of *AlkB* gen. *AlkB*-harboring bacteria were significantly stimulated in the BIOG biofilm population. Further, this fact is relevant since the *AlkB* gene is one of the key participants in PE degradation. The *AlkB* gene abundance analysis revealed a significant effect of the culture time and the consortium type. It was concluded that tailored indigenous communities of polymer and hydrocarbon-degrading species can degrade naturally weathered PE films before they are converted into microplastics in the marine environment [76].

Another study using 16S rRNA microbiome profiling of surface and sediment plastic-associated microbial biofilms [77] revealed *Bacteroidetes* and *Gammaproteobacteria* as the main bacterial groups. This work showed that the bacterial community composition depends on the plastic’s nature. For instance, enrichment of the *Alcanivorax*, *Marinobacter*, and *Arenibacter* genera occurred on Low-density PE (LDPE) and PET. Also, these authors demonstrated the ability of *Alcalinovorax borkumensis* to degrade LDPE [77]. *Pseudomonas knackmussii* N1-2 and *P. aeruginosa* RD1-3 have also been reported as responsible for PE degradation [78]. Other genera and species are reported as capable of degrading LDPE [79] (Table 1).

**Table 1:** Microorganisms degrading polymers obtained from fossil resources.

Polymer/Acronym	Chemical Structure	Degrading Microorganism	Reference
Poly(ethylene adipate)/PEA	[-OCH_2_CH_2_OOC(CH_2_)_4_CO-]_n_	*Penicillium* sp. strain 14-3, *Penicillium* sp. (ATCC 36507); *Pullularia* sp.	[33,68]
Poly(ethylene succinate)/PES	[-O(CH_2_)_2_OOC(CH_2_)_2_CO-]_n_	*Penicillium* sp. strain 14-3, *Bacillus pumilis*, *Aspergillus clavatus*, *Streptomyces s*p., *Actinomadura* sp., *Thermoactinomyces* sp. *Saccharomonospora* sp.	[33]
		*Pseudomonas* sp. AKS2, *Microbispora* sp., *Bacillus subtilis*, *Paenibacillus amylolyticus*, *Rhizopus delemar*	[58]
Poly(butylene succinate)/PBS	[-O(CH_2_)_4_OOC(CH_2_)_2_CO-]_n_	*Penicillium* sp. strain 14-*Microbispora rosea*, *Excellospora japonica*, *Excellospora viridilutea*, *Firmicutes*, *Proteobacteria*, *Streptomyces* sp.	[33]
Poly(butylene adipate)/PBA	[-O(CH_2_)_4_O_2_C(CH_2_)_4_CO-]_n_	*Penicillium* sp. strain 14-3	[33]
Poly(ethylene adipate)/PEA and Poly(-ε-caprolactone)/PCL	[-OCH_2_CH_2_OOC(CH_2_)_4_CO-]_n_ [-OCH_2_CH_2_CH_2_CH_2_CH_2_CO-]_n_	*Rhizopus arrhizus*, *R. delemar*, *Achromobacter* sp., *Candida cylindracea*, *Aspergillus*, *Aureobasidium*, *Penicillium*, *pullularia.*	[33,68]
PCL	[-OCH_2_CH_2_CH_2_CH_2_CH_2_CO-]_n_	*Aspergilus* sp. ST-01, *Penicillium* sp. (ATCC 36507), *Firmicutes*, P*roteobacteria*, *Clostridium* sp., *Aspergillus flavus*, *Penicillium funiculosum.* *Arcobacter thereius*	[33,58]
		*Shewanella* sp., *Moritella* sp., *Psychrobacter* sp., *Pseudomonas* sp.	[75]
PBS, PCL, and PLA		*Saccharothrix JMC9114*, *Kibdelosporangium aridum JMC7912*, *Actinomadura keratinilytica T16-1*, *Amycolatopsis thailandensis PLA07*, *Streptomyces bangladeshensis 77T-4*, *Streptomyces thermoviolaceus 76T-2*, *Aureobasidium* sp., *Chaetomium* sp., *Rhizopus* sp., *Thermoascus aurantiacus*, *Cryptococcus* sp. *S-2.*, and *Pseudozyma anctarctica.*	[68]
		*Cryptococcus laurentii*, *Clostridium botulinum*, *Alcaligenes faecalis.* *Bacillus brevis*, *Clostridium botulinum*, *C. acetobutylicum*, *Amycolatopsis* sp., *Fusarium solani*, *Aspergillus flavus*, *Tenacibaculum* sp., *Alcanivorax* sp., and *Pseudomonas* sp.	[58]
PCL and PLA		*Arcobacter thereius*, *Methanobacterium petrolearium*, *Methanosaeta concilii.*	[80]
		*Chaetomium globosum* ATCC 16021	[66]
		*Streptoverticillium kashmeriense* AF1	[71]
Poly(β-propiolactone)/PPL	[-OCH_2_CH_2_CO-]_n_	*Bacillus* sp., *Acidovorax* sp., *Variovorax paradoxus*, *Sphingomonas paucimobilis*, and *R. delemar*	[33]
Poly(propylene succinate)/PPS and poly(butylen tereftalate/PBT	[O(CH_2_)_3_O_2_CCH_2_CH_2_CO]_n_ [OOCC_6_H_4_COO(CH_2_) _4_]_n_	*R. delemar*	[33]
AAC’s		*Thermobifida fusca*	[33]
Poly(ethylene)/PE	[–CH_2_CH_2_–]_n_	*Lysinibacillus xylanilyticus*, *Aspergillus niger*, *A. versicolor*, *A. flavus*, *Cladosporium cladosporioides*, *Fusarium redolens*, *Fusarium* sp. AF4, *Penicillium simplicissimum* YK, *P. pinophilum*, *P. frequentans*, *Phanerochaete chrysosporium*, *Verticillium lecanii*, *Glioclodium virens*, *Mucor circinelloides*, *Acremonium kiliense*, *Gordonia* sp., *Nocardia* sp., *Staphylococcus* sp., *Streptococcus* sp., *Micrococcus* sp., *Streptomyces* sp., *Rhodococcus* sp., *Proteus*sp., *Listeria*sp., *Vibrio* sp., *Brevibacillus* sp., *Serratia* sp., *Diplococcus* sp., *Moraxella* sp., *Penicillium* sp., *Arthrobacter* sp., *Aspergillus* sp., *Phanerochaete* sp., *Chaetomium* sp., *Gliocladium* sp., *Mucor rouxii*, *Methylobacter* sp., *Nitrosomonas* sp. AL212, *Nitrobacter winogradskyi*, *Burkholderia* sp., Me*thylococcus capsulatus*, *Methylocystic* sp., *Methylocella* sp., *Streptomyces coelicoflavus* 15399, *B. thuringiensis*, *Stenotrophomonas pavanii*, *Paecilomyces lilacinus*, *Lysinibacillus fusiformis*, *Bacillus cereus*, *Bacillus mycoides*, *Avicennia marina*, *Pseudomonas citronellolis*, *Burkholderia seminalis*, *Lasiodiplodia theobromae*, *Pseudomonas* sp. AKS2.	[58]
		*Aspergillus japonicus*	[59]
		*Pseudomonas knackmussii*, *Pseudomonas aeruginosa.*	[78]
		*Brevibacillus parabrevis*, *P. citronellolis*, *Acinetobacter baumannii.*	[79]
		*Trametes versicolor*, *Rhodococcus ruber.*	[65]
		*Brevibacillus borstelensis*, *Acinetobacter baumannii*, *Bacillus amyloliquefaciens*, *B. brevis*, *B. cereus*, *B. circulans*, *B. halodenitrificans*, *B. mycoides*, *B. pumilus*, *B. sphaericus*, *B. thuringiensis*, *Arthrobacter paraffineus*, *A. viscosus*, *Microbacterium paraoxydans*, *Nocardia asteroides*, *Micrococcus luteus*, *M. lylae*, *Paenibacillus macerans*, *P. aeruginosa*, *P. fluorescens*, *Rahnella aquatilis*, *Ralstonia spp.*, *R.ruber*, *R. rhodochrous*, *Rhodococcus erythropolis*, *Staphylococcus cohnii*, *S. epidermidis*, *S. xylosus*, *Stenotrophomonas* sp., *Streptomyces badius*, *S. setonii*, *S. viridosporus.*	[22]
		*Penicillium pinophilum*, *A. cremeus*, *A. candidus*, *A. niger*, *A. nidulans*, *A. glaucus*, *A. flavus.*	[68]
Poly(urethane) PUR		*Pseudomonas chlororaphis* ATCC 55729, *Comamonas acidovorans* TB-35, *Chaetomium globosum*, *Aspergillus terreus*, *Fusarium solani*, *Candida ethanolica*, *Curvularia senegalensis*, *Aspergillus fumigatus*, *A. niger*, *A*. *flavus*, *Emericella*sp., *Lichthemia*sp.,*Thermomyces* sp., *Corynebacterium* sp., *Neonectria*sp., *Plectosphaerella*sp., *Phoma*sp., *Nectria*sp., *Alternaria*sp., *P. aeruginosa*, *Bacillus* sp., *Aspergillus foetidus*, *Pestalotiopsis microspora*, *Acinetobacter gerneri*, *Aspergillus terreus*, *Aspergillus fumigatus*, *Aspergillus flavus*, *Fusarium solani*	[58]
		*Lasiodiplodia* sp. E2611A., *Bionectria* sp., *Pestalotiopsis microspora*	[59]
		*Spicaria* sp. *Alternaria solani.*	[68]

**Table 2:** Microorganisms degrading polymers obtained from renewable resources.

Polymer/Acronym	Chemical Structure	Degrading Microorganisms	Reference
Poly(hydroxyalkanoates)/PHAs		*Acinetobacter calcoaceticus*, *Arthrobacter artocyaneus*, *Bacillus aerophilus*, *Bacillus megaterium*, *Brevibacillus agri*, *Brevibacillus invocatus*, *Chromobacterium violaceum*, *Cupriavidus gilardii*, *Mycobacterium fortuitum*, *Ochrobactrum anthropi*, *Staphylococcus arlettae*, *Staphylococcus haemolyticus*, *Staphylococcus pasteuri*, *Pseudomonas acephalitica*, *Rodococcus equi*, *Bacillus cereus*, *Bacillus mycoides*, *Gordonia terrari*, *Microbacterium paraoxidans*, *Nocardiopsis* sp., *Streptomyces* sp., *Burkholderia* sp., *Gongronella butleri*, *Penicillium* sp., *Acremonium recifei*, *Paecilomyces lilacinus*, *Trichoderma pseudokoningii*.	[64]
PHAs/PCL/not PLA		*Terrabacter tumescens*, *Terracoccus luteus*, *Brevibacillus reuszeri*, *Agrobacterium tumefaciens*, *Duganella zoogloeoides*, *Ralstonia eutropha*, *Ralstonia pickettii*, *Matsuebacter chitosanotabidus*, *Roseateles depolymerans*, *Rhodoferax fermentans*, *Variovorax paradoxus*, *Acinetobacter calcoaceticus*, *Acinetobacter junii*, *Pseudomonas pavonaceae*, *Pseudomonas rhodesiae*, *Pseudomonas amygdali*, *Pseudomonas veronii.*	[74]
Poly(3-hydroxybutyrate)/P3HB	[-(OCH_3_)CHCH_2_CO-]_n_	*S. kashmirens*, *Bacillus* sp., *Streptomyces* sp.	[71]
		*Comamonas testosterone*, *Pseudomonas lemoignei*, *Pseudomonas stutzeri*, *Acidovorax faecalis*, *Aspergillus fumigatus*, *Variovorax paradoxus*, *Sphingomonas paucimobilis*, *Amycolatopsis* sp. HT-6, *Alcaligenes faecalis*, *Ilyobacter delafieldii*, *Penicillium funiculosum*, *Schlegelella thermodepolymerans*, *Paecilomyces lilacinus*, *Fusarium* sp., *Trichoderma* sp., *Alternaria* sp., *Aspergillus oryzae.*	[33,58]
		*Nocardiopsis aegyptia*	[67]
		*Acremonium* sp., *Cladosporium* sp, *Debaryomyces* sp., *Emericellopsis* sp., *Eupenicillium* sp., *Fusarium* sp., *Mucor* sp., *Paecilomyces* sp., *Penicillium* sp., *Pullularia* sp., *Rhodosporidium* sp., *Verticillium* sp.	[68]
P3HB and Poly(ethylene succinate)/PES	[-(OCH_3_)CHCH_2_CO-]_n_ ([-O(CH_2_)_2_OOC(CH_2_)_2_CO-]_n_	*Actinomadura* sp., *Microbispora* sp., *Streptomyces* sp., *Thermoactinomyces* sp., *Saccharomonospora* sp., *Aspergillus ustas*.	[33]
Poly(lactic acid)/PLA	[-O(CH_3_)CHCO-]_n_	*Amycolatopsis* sp., *Saccharotrix* sp., *Tritirachium album*.	[33]
		*Streptomyces bangladeshensis.*	[70]
		*Amycolatopsis* sp., *Penicillium roqueforti*, *Bacillus brevis*, *Rhizopus delemar.*	[71]
		*Fusarium moniliforme*, *Mesorhizobium* sp., *Actinomadura keratinilytica* NBRC 104111, *Bacillus amyloliquefaciens*, *Pleurotus ostreatus*, *Cryptococcus* sp. S-2, *Aneurinibacillus migulanus*, *Pseudomonas tamsuii*, *Thermopolyspora* sp., *Thermomonospora* sp., *Paecilomyces* sp.	[58]
		*Bordetella petrii*, *Penicillium roqueforti*, *Kibdelosporangium aridum* JMC7912, *Actinomadura keratinilytica* T16-1, *Amycolatopsis thailandensis* PLA07	[68]
Poly(3-hydroxybutyrate-*co*-3-hydroxyvalerate)/P3HB-*co*-P3HV		*Clostridium botulinum*, *C. acetobutylicum*, *Streptomyces* sp. SNG9	[58]
		*Streptoverticillium kashmeriense* AF1	[71]

### 4.1. Degradation of Copolymers by Individual or Consortium Microorganisms

Polymer blends reduce the cost of materials while specific properties are obtained, and the degradation rate may be modified. However, the mixture of a biodegradable polymer with another non-biodegradable polymer may decrease or inhibit the degradation of the biodegradable component. *N. aegyptia*, a P3HB degrader, also degrades the copolymer p(3-hydroxybutyrate-*co*-3-hydroxyvalerate) [p(3HB-*co*-10-20%3HV)]. Further, a greater efficiency in the degradation of the copolymer P(3HB-*co*-20%3HV) was observed in comparison with the pure P3HB and the P(3HB-*co*-10%3HV) copolymer [67].

In the AACs biodegradation research, a lipase from *R. delemar* hydrolyzed some copolyesters constituted by PCL and an aromatic polyester such as PET or poly(butylene terephthalate) (PBT). Furthermore, it was demonstrated that the biodegradability of these copolyesters decreases with increasing content of aromatic polyester [81]. Other AACs synthesized from 1,4 butanediol, adipic acid, and terephthalic acid are attacked by *Termobifida fusca*, a bacterium isolated from compost, with a rate of AACs degradation 20 times higher than that observed in tests of typical compost. An inducible thermophilic hydrolase for AACs and esters was discovered in *T. fusca,* designated TfH, whose optimum temperature is 65 °C, and it has an active site with a conserved amino acid sequence typical of serine hydrolases. The degradative characteristics of TfH differ significantly from conventional lipases since lipases require an activation surface and hydrolyze ester bonds only at a hydrophobic surface. In contrast, TfH hydrolyzes dissolved esters, a typical esterase activity, suggesting that TfH combines features of lipase and esterase [32,82]. Lipases from *Candida antarctica* and *Pseudomonas* sp. do not significantly attack the PET film, while TfH at 55 °C dissolved a 100 μm film in three weeks. The unexpected degradation of PET by TfH is due to several factors. First, the degradation temperature (55 °C), at which TfH is active and stable for a sufficient period to degrade the polyester, leads to a smaller Δ*T_mt_* and, therefore, greater flexibility of the chains in the PET crystalline domains. Also, the difference between the degradation and melting temperatures is still very high, resulting in a low degradation rate. Therefore, significant enzymatic degradation of PET could be carried out due to the low crystallinity (less than 10%) [32]. A synergistic effect on the PET degradation rate was observed in a study with high crystallinity PET using a synergistic microbe–enzyme treatment composed of *Microbacterium oleivorans* JWG-G2 and *T. fusca* cutinase. The analysis of the *M. oleivorans* genome revealed the presence of extracellular hydrolase-coding genes, including three carboxylesterases, one esterase, and one lipase [83].

In rumen microbiology, research is underway to assess the ability of ruminal microorganisms to degrade PET. Since thousands of microorganisms can degrade polymers such as cellulose and starch in the rumen, it has been hypothesized that PET-degradative organisms could also exist. It is estimated that under anaerobic conditions, the degradation of the PET basic unit, the benzene ring, would require a minimum of 70 days. Even when the rumen food only lasts 72 h, it was found that rumen microorganisms can degrade PET by breaking the benzene ring, transforming it into terephthalic acid, and using it as an energy source [84]. A metagenomic study of rumen microbiota showed the presence of *Proteobacteria* as the predominant group, followed by *Bacteroidetes*, *Firmicutes*, and *Actinobacteria*. *Pseudomonas veronii* was the dominant species, followed by *Acinetobacter* sp.; eukaryotes belonging to the clade Opisthokonta, including metazoans, fungi, and other microorganisms such as Choanoflagellida, Ichthyosporea, Nucleariida, and *Capsaspora* sp., were also found. Among fungi, Phyla Ascomycota and Basidiomycota, both cellulose degraders, and Mucormycota were detected [85]. *Ideonella sakaiensis* 201-F6, capable of using PET as an energy and carbon source, was isolated in another study [86]. From PET-debris-contaminated environment samples, these authors searched for microorganisms using low-crystallinity PET for growth. They found a microbial consortium degrading the PET film at a rate of 0.13 mg/cm^2^/day at 30 °C. Further, 75% of the degraded PET film was mineralized into CO_2_ at 28 °C. When the consortium lacked *I. sakaiensis*, it also lost the PET degradation capability [86].

### 4.2. Archaea, Cyanobacteria, and Algae Degrading Plastic: Discovering Unknown Abilities from Old Earth Inhabitants

The archaea *Methanosaeta concilii* and *Methanobacterium petrolearium* were found to be involved in the anaerobic biodegradation of PCL and PLA [80]. Approximately 0.9% of the ruminal microbiome corresponded to Archaea, the bacterial group responsible for rumen methanogenesis, including Euryarchaeota, within which the most represented genus was *Methanobrevibacter* [85].

Using metagenomics, the microbial communities on marine PP and PE debris were studied [61]. They found photosynthetic filamentous cyanobacteria *Phormidium* sp., *Rivularia* sp., and diatoms such as *Navicula* sp., *Nitzschia* sp., *Sellaphora* sp., *Stauroneis* sp., and *Chaetoceros* sp. Of these, the first three are biofilm formers. Also, DNA sequences from radiolarians were identified in both plastic types. Some OTUs were found in both plastics but not in the water, e.g., *Phormidium* sp., a hydrocarbon-degrading bacterium, and *Pseudoalteromonas* sp., a genus associated with marine algae. Members of the Hyphomonadaceae family were unique to plastic marine debris, comprising 8% of the OTUs on PP. Further, this hydrocarbon-degrading group can be methylotrophic. This study detected other potential hydrocarbon-degrading OTUs: *Haliscomenobacter* and *Devosia*, associated with hydrocarbon-contaminated soils, and *Oceaniserpentilla*, one of the major taxa from OTUs related to the Deepwater Horizon oil spill. These authors’ findings suggested that the consortia may be acting in concert to metabolize the plastic [61]. A research reported the ability of *Scenedesmus dimorphus*, *Anabaena spiroides*, and *Navicula pupula*, a green alga, a blue–green alga, and a diatom, respectively, to biodegrade PE sheets (at 27 °C) [87]. The microalgae were isolated from the domestic PE bags dumped in the suburban water bodies. The highest average percentage of degradation, 8.18%, was observed with *A. spiroides*, presumably due to the ability of this filamentous blue–green alga to form biofilms and cavities on the surface of the PE sheets. All the microalgae colonized more efficiently in the low-density PE sheets than in the high-density PE sheets. The authors found that microalgae colonies predominated on the surface of PE bags due to accessibility to nutrients, water, and sunlight [87]. Due to their abundance, microalgae represent a promising resource in the field of polymer degradation.

### 4.3. Plastic-Degrading Microorganisms Living in the Waxworm’s Gut: A New Source for Microbial Enzyme Searching

Several studies have reported the biodegradation of polyethylene (PE) by microorganisms in the gut of insect larvae, particularly the larval stage of *Plodia interpunctella* and *Achroia grisella* [88,89]. In another study with larvae [90], researchers found that mealworms (the larvae of *Tenebrio molitor* Linnaeus) chew and digest Styrofoam, a poly(styrene) product. The Styrofoam was efficiently degraded in a retention time of less than 24 h. The authors found that 47% of the ingested Styrofoam carbon was converted into CO_2_, and 49.2% was egested as feces, with a small fraction (ca. 0.5%) incorporated into biomass. The finding of polymer-degrading microorganisms in waxworms’ guts represents an unexpected alternative to plastic degradation [90].

According to the above-mentioned, a variety of microorganisms can degrade plastics. In nature, there are abundant natural polymers: cellulose, starch, chitin, chitosan, pullulan, collagen, gelatin, and alginates [34]. Consequently, diverse microorganisms can degrade natural polymers [58]. Therefore, microorganisms on Earth first degraded natural polymers, developing the enzymatic degradation machinery necessary for the arrival of synthetic polymers.

However, a recent critical review pointed out that most studies on PE biodegradation lack key methodological elements to confirm microbial assimilation or mineralization, e.g., isotope-labeled polymers, quantitative labeled CO_2_ measurements, and adequate controls. According to the author, there is no convincing evidence that PE or PVC can be biodegraded to CO_2_, and more rigorous approaches are needed to irrefutably demonstrate such processes [60]. In line with these methodological standards, a study was conducted to investigate the potential bioassimilation of PE by *Galleria mellonella* larvae using fully deuterated PE and infrared microspectroscopy [89]. Although the authors observed polymer fragmentation and weak oxidation within the gut, their high-resolution spectral histology showed no evidence of deuterium incorporation into larval tissues [89]. These results suggest that PE was physically and chemically modified but not metabolized or bioassimilated by the larvae, reinforcing the non-biodegradability of PE stated by [60].

## 5. Genetic Basis of Plastic Degradation

For plastic degradation, the microorganisms use diverse enzymatic machinery: glucosidases, catalases, proteases, cutinases, serine hydrolases, lipases, manganese peroxidases [91], esterases, laccases, hydroquinone peroxidase, alkane hydroxylase, aryl acyl amidase, nylon hydrolase [65], urease, papain, and subtilisin [58]. In some cases, plastic degradation occurs through multiple enzymes and metabolic pathways [92]. Regarding the genes behind the microbial degradation process, knowledge is still scarce. An early study investigated gene-encoding enzymes involved in the degradation of synthetic polymers, particularly nylon oligomers [93]. They reported three genes (*nylA*, *nylB*, and *nylC*) that encode hydrolases that are able to degrade nylon oligomers. Another study reported a phthalate degradation operon in the *Mycobacterium vanbaalenii* PRY1 genome [94]. Five tandem genes were identified: *phtAa* (phthalate dioxygenase large subunit), *phtAb* (small subunit), *phtB* (phthalate dihydro diol dehydrogenase), *phtAc* (phthalate dioxygenase ferredoxin subunit), and *phtAd* (phthalate dioxygenase ferredoxin reductase), as well as a putative regulatory gene (*phtR*). The operon is conserved in diverse polycyclic aromatic hydrocarbon-degrading *Mycobacterium* spp., isolated from various geographical locations [94].

Studies with *A. oryzae* demonstrated its ability to degrade PBSA by the cutinase CutL1. Additionally, the presence of PBSA induces the *rolA* gene overexpression in *A*. *oryzae*. The hydrophobin RolA binds to the solid PBSA surface and recruits CutL1, stimulating the hydrolysis of PBSA [95]. Another hydrophobic surface-binding protein, HsbA from *A. oryzae*, promotes PBSA degradation through interaction with CutL1 on the PBSA surfaces. HsbA is not classified as a hydrophobin. Thus, *A. oryzae* uses several surface-active proteins to degrade PBSA [69]. Another research [96] reported the *Pseudomonas* sp. E4, a strain capable of degrading low molecular weight PE (LMWPE). These authors amplified an alkane hydroxylase gene (*AlkB*) from this strain and cloned it into pUC19. Then, they transformed *E. coli* BLZ21 with the plasmid harboring the *AlkB* gene. The recombinant *E. coli* BLZ21 acquired the ability to degrade LMWPE, whereas the untransformed *E. coli* BLZ21 could not degrade the polymer. This study demonstrated the essential role of the *AlkB* gene, which encodes alkane 1-monooxygenase, in LMWPE degradation [76,96]. In contrast, when the lignin-degrading fungi *Phanerochaete chrysosporium* and *Trametes versicolor* were under different nutritional conditions, the primary enzyme involved in PE degradation was a Manganese peroxidase [59].

In another work, [97] a 200 kb plasmid was isolated in the polyethylene-degrading *Bacillus megaterium* strain B1 bacteria, designated as the PE plasmid. The authors showed that this plasmid facilitates plastic degradation; when the plasmid was removed, the bacteria lost their ability to degrade plastic. Moreover, these authors transformed *E. coli* DH5α strains with that plasmid, and the *E. coli* strains acquired the ability to degrade polyethylene [97]. In *Pseudozyma antarctica* JCM 10317, a strain with outstanding degradative activity, the PaE enzyme encoded by the gene *PaCLE1* was isolated. This enzyme showed an identity of 61 to 68% with cutinase-like enzymes and can degrade biodegradable plastics, PBS, PBSA, Poly(ε-caprolactone), and PLA [98]. In *C. acidovorans* TB-35, the gene *PudA* is the key gene encoding the enzyme PUR esterase, enabling this bacterium to degrade polyester-PU [58]. The bacterium *I. sakaiensis* 201-F6 was reported to break down PET and utilize it as a major energy and carbon source [86]. The gene *ISF6_4831* is one of the genes encoding the PET hydrolases, which break the ester bonds of PET, resulting in the production of mono (2-hydroxyethyl) terephthalate (MHET) as a first degradation product. Other genes encoding PET hydrolases were reported in other microorganisms, such as *Saccharomonospora viridis*, designated as the *Cut190* gene [99], and *Thermomonospora curvata*, denominated the *Tcur_1278 gene* [100]. Recently, the green algae *Chlamydomonas reinhardtii* was transformed to produce PETase, and the expression of the *PETase* gene was evaluated [101]. Two strains were transformed using the plasmid pBR9_PETase_Cre, and as a result, five of the clones of CC-124 transformants showed expression for PETase after five days of cultivation.

Considering the ubiquity of plastics in ecosystems, together with the enormous genetic and metabolic diversity of microorganisms, it is likely that microorganisms in various habitats have developed different capacities to break down and use plastics. The genes involved in the degradation of plastics identified so far may represent only a tiny portion of the genes involved in the depolymerization of plastics in the environment. Therefore, developing high-throughput screening approaches, such as metagenomics and proteomics, could accelerate and facilitate the discovery of new plastic-degrading microorganisms and enzymes [102].

Esterases capable of hydrolyzing poly(diethylene glycol adipate) (poly DEGA) and synthetic poly(butylene adipate-*co*-butylene terephthalate) copolyester (PBAT) were identified in metagenomic libraries constructed from soil compost and *Sphagnum moss*, respectively [103]. In another study, an analysis of terrestrial and marine metagenomes revealed a wide distribution but a low frequency of genes encoding PET hydrolytic enzymes, indicating the slow evolution of indigenous microorganisms to use anthropogenic PET [104]. Many gene-encoding enzymes capable of depolymerizing different plastic materials have been recovered from a wealth of environmental metagenome samples [105]. On the other hand, a previous study [106] reported that the marine bacterium *Bacillus* AIIW2 could degrade PET. The comparison of the transcriptomic profile of the AIIW2 strain suggested that the hydrolytic enzymes, such as carboxylesterase and aldehyde dehydrogenase had a key role during the incubation of the PET film with the bacteria. The authors observed that the carboxylesterase gene (*EME73206.1*) was crucial in the degradation process. However, most published studies focused primarily on biodegradation by wild-type strains isolated directly from plastic-contaminated areas. Although the results indicate positive effects on the degradation of plastics, it has not been enough to reduce plastic contamination, so the plastic degradation capacity must be improved. Therefore, to improve the biodegradation rate of plastic, more efficient production of enzymes with greater activity on specific materials is required, which can be achieved using genetic or metabolic engineering. Genetic manipulation makes it possible to produce mutant enzymes with improved catalytic activity and thermostability in plastic hydrolysis. Conversely, bioinformatics has also played an essential role in accelerating plastic biodegradation research. In this sense, an algorithm for identifying PET hydrolases synthesized by different bacterial phyla in various environments was developed [104]. The authors identified over 800 potential candidates, originating mainly from *Actinobacteria*, *Proteobacteria*, and *Bacteroidetes*. An online database [107] (PMBD: Plastics Microbial Biodegradation Database) was constructed, compiling more than 900 microorganisms, 8000 predicted enzyme sequences, and 79 confirmed genes involved in the biodegradation of plastic [107]. Recently, a research group [108] developed the PlasticDB web application, which comprises a database of microorganisms and proteins reported to biodegrade plastics. The database was implemented with several analytical tools that accept inputs, including genes, genomes, metagenomes, transcriptomes, metatranscriptomes, and taxa table data. These bioinformatic tools can be used to identify microorganisms and proteins that may be involved in plastic biodegradation, compare the genetic potential for plastic biodegradation through datasets, and analyze plastic biodegradation pathways [108].

## 6. Concluding Remarks and Future Trends

Plastics are essential in our daily lives, but they represent a severe problem due to the large amounts of plastic waste that modern society generates annually worldwide. Since nature is rich in polymers, it is not surprising that microorganisms capable of degrading natural polymers have evolved. Further, diverse plastic-degrading microorganisms have been reported in gardens, farms, beaches, compost, polar waters, mangrove soils, ruminal, and marine and freshwater environments. The slow rate of plastic degradation in ecosystems has been of great concern in recent decades. Biotechnological advances have enabled the production of biodegradable plastics that, unlike petroleum-based plastics (whose degradation can take centuries), can be degraded in a few months, under appropriate conditions. However, some studies have raised concerns because they have not been able to demonstrate the bioassimilation and mineralization of some polymers by microorganisms.

Due to their non-toxic degradation products, starch-based polymers have emerged as promising alternatives, particularly for agricultural applications and food packaging, both activities with a high impact on ecosystems due to the number of plastics used. However, recent toxicological findings in murine models suggest that starch-based microplastics may induce metabolic and tissue-level alterations comparable to those caused by conventional plastics. These results highlight the importance of thoroughly evaluating the safety of bioplastics before their widespread application, especially in food-related systems. Research into the microbial degradation of plastics remains a key strategy to mitigate plastic pollution. Ongoing efforts include the study of microbial diversity, the discovery of hydrolytic enzymes, the engineering of microbial consortia, and the genetic manipulation of bacterial strains to generate more efficient plastic-degrading bacteria and to obtain strains producing bioplastics. Continued research in this area is essential to developing safer and more effective biodegradable plastics and clarifying the potential and limitations of microbial degradation as a large-scale solution to plastic pollution.

## Data Availability

No new data were created or analyzed in this study.
